# Bruno da Longobucco (da Longoburgo): The first academic surgeon in the Middle Ages

**DOI:** 10.3389/fsurg.2022.1025987

**Published:** 2022-12-26

**Authors:** Francesco Pata, Cataldo Linardi, Richard R. Brady, Gianluca Pellino, Giancarlo D’Ambrosio, the Bruno da Longobucco study group, Marialuisa Vennari

**Affiliations:** ^1^ General Surgery Unit, Nicola Giannettasio Hospital, Corigliano-Rossano, Italy; ^2^ Sapienza University, Rome, Italy; ^3^ Operating Theatre Department Nicola Giannettasio Hospital, Corigliano-Rossano, Italy; ^4^ Newcastle Centre for Bowel Disease Research Group, Newcastle Hospitals, Newcastle, United Kingdom; ^5^ Department of Advanced Medical and Surgical Science, Università degli Studi della Campania ‘Luigi Vanvitelli’, Naples, Italy; ^6^ Colorectal Surgery, Vall d'Hebron University Hospital, Barcelona, Spain; ^7^ Department of General Surgery, Surgical Specialties and Organ Transplantation, Sapienza University, Rome, Italy

**Keywords:** history of surgery, Bruno da Longobucco, Longoburgo, surgeon, surgery, Middle Ages, medieval, historical overview

## Abstract

Bruno da Longobucco (1200–1286) was born at the turn of the 13th Century in Longobucco (Calabria, Italy), at that time named Longoburgo. He was the first academic surgeon of the Middle Ages, a period when surgery was disregarded by mainstream physicians and was the practice of barbers, charlatans and phlebotomists. After training at the medical school of Salerno and the University of Boulogne, he was one of the founders of the University of Padua and became the first Professor of Surgery. His books *Chirurgia Magna* and *Chirurgia Parva*, were ones of the most disseminated surgical texts of the Middle Ages and it is argued helped surgery regain its reputation. Despite his importance to late medieval period, he has been essentially overlooked in the records of the history of surgery. Currently, there are no articles in English about his life indexed on PubMed, Scopus or Embase. One solitary article on Bruno's life and influence was published in 1960s in a small journal in Italian, but this is no longer active and there is no electronic means to access the original article. The aim of this article is to provide education and rediscovery of the impact of this critical figure, his works and his historic role to the development and renaissance of surgery for contemporary surgeons.

## Introduction

In the early Middle Ages (6th–10th centuries), medicine was approached as a theoretic activity. The Platonic bias “theory is higher than practice” promoted the development of a purist and speculative discipline which led to a reduced emphasis on less intellectual pursuits, such as manipulative techniques (1). In this cultural change, manual activity such as surgery was regarded as unworthy of the practice of physicians (2). Operations were often unsuccessful due to the absence of anesthesia and antisepsis, with high mortality and complication rates, contributing to a rising skepticism about the value of this approach. In absence of any formal training or accreditation, surgical practice was often undertaken by groups of empirics, barbers and charlatans, or elsewhere by clergy in monasteries (3).

There is a common believe amongst history scholars that medieval religion prohibited the study of anatomy and surgery and regarded it as blasphemous, as summarized by the quote “*Ecclesia abhorret a sanguine*” (“Church refuses blood”). However, it is felt that this is a apocryphal exaggeration of the situation, as this phrase was never actually found in any official documents from the time and is attributed to just one reference by François Quesnay in his “History of surgery in France”, published in the 18th century (4). Indeed, the Church undertook major efforts to avoid the loss of ancient medical heritage and texts, preserving and painstakingly transcribing medical manuscripts from Greek and Latin authors in local monasteries thanks to the work of monks, called amanuenses.

With the translation of Arabic authors as Avicenna, Haly Abbas, and Albucasis through the 10th and 11th centuries and a renewed academic interest in the insights and learning from Galen in the 11th and 12th century, authors who explored the practice of surgery in their texts, the profile and reputation of surgery underwent a revival amongst physicians (5).

**Figure 1:**
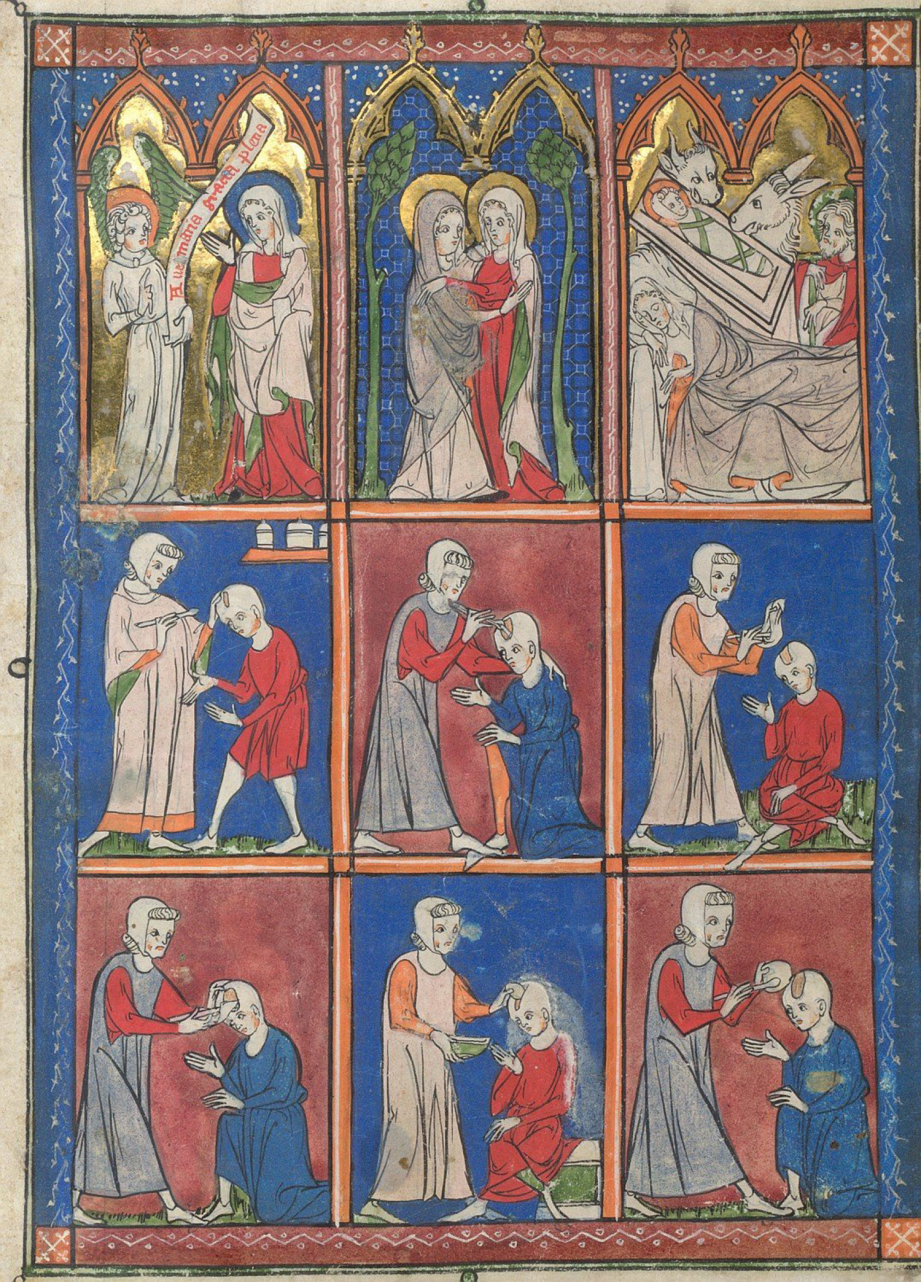
Illustrations of surgical procedures from a French compendium of Roger Frugard's *Chirurgia* (from the British library, London UK. https://www.bl.UK/#).

Italy was the cradle of this renaissance in surgery (6). Many texts were rediscovered through the work of the Medical School of Salerno, where Surgery was already taught in the 10th century. The first book of surgery (Figure 1), Chirurgia (known also as *Post Mundi Fabricam*) was written by Roger Frugardi (also known as Ruggero dei Frugardi) around 1180 (7). The core approach of Roger Frugardi's text was essentially an operative manual describing the oral lessons of surgery taught at the medical school in Salerno, inspired by Latin and Byzantine ancient literature. This text and a subsequent revised commentary by one of his pupils, Rolando da Parma (8) was disseminated widely to other parts of Italy and abroad, especially France, where surgery was taught, although without any formal recognition, in some schools.

In 1231, Frederick II promulgated the *Constitutions of Melfi* for his Kingdom of Sicily. In this document, a formal examination by the masters of Salerno Medical School was required in order to practice medicine (9). This academic and political movement towards a formal regulation of Medicine and other disciplines promoted the institutionalization of pre-existing schools and student corporations (named *Universitas*) with the foundation of the first universities, such as Oxford (1164), Naples (1224), Padua (1222), Boulogne (1088), Salamanca (1212), Paris (1138).

Despite this, Surgery remained through this era a “second-class” subject (10). In the 13th century, in Montpellier, formally trained doctors were considered competent also in surgery, but those who trained only in Surgery could not offer any medical therapy, with separate examinations created to avoid scandal (5). In the 1400s in Padua, there was a separate degree for Surgery offered, but no student completed it in 30 years, from 1405 to 1435. This inequality between the disciplines remained there well until the 16th century: for instance, the Professor of Anatomy and Surgery had a salary of 70 florins compared the 1,000-florin salary of a Professor of Medical Practice (6).

**Figure 2:**
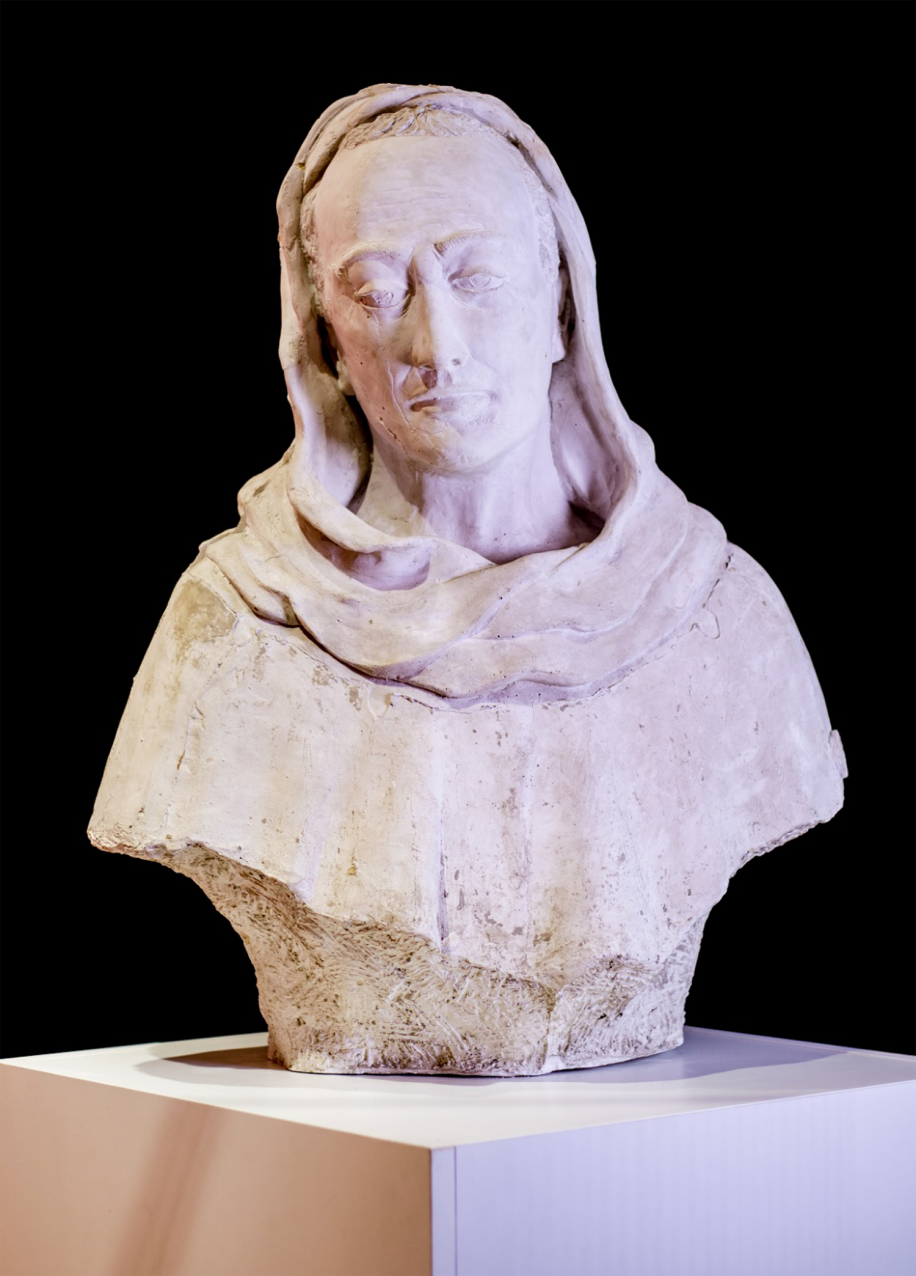
Bruno da Longobucco (Bruno da Longoburgo), in the free interpretation of the local artist Thomas Pirillo (1952–2021). (Courtesy of Proloco del Comune di Longobucco).

The remainder of this article will focus on the critical events of the 1200s, which drove an irreversible process of renaissance in the practice of surgery and its incorporation into mainstream medical practice with the critical involvement of the namesake protagonist of this article, Bruno da Longobucco (Figure 2).

## Life and works

Currently, no article in English about Bruno is indexed on PubMed, Scopus, Embase. The only article recorded on PubMed focusing on Bruno's impact was published in 1960s in a small journal in Italian language, now since no longer operating and without the opportunity to download the article electronically (11). Moreover, many previous literary sources have published only in Italian language, making a forensic overview of Bruno da Longobucco challenging.

**Figure 3:**
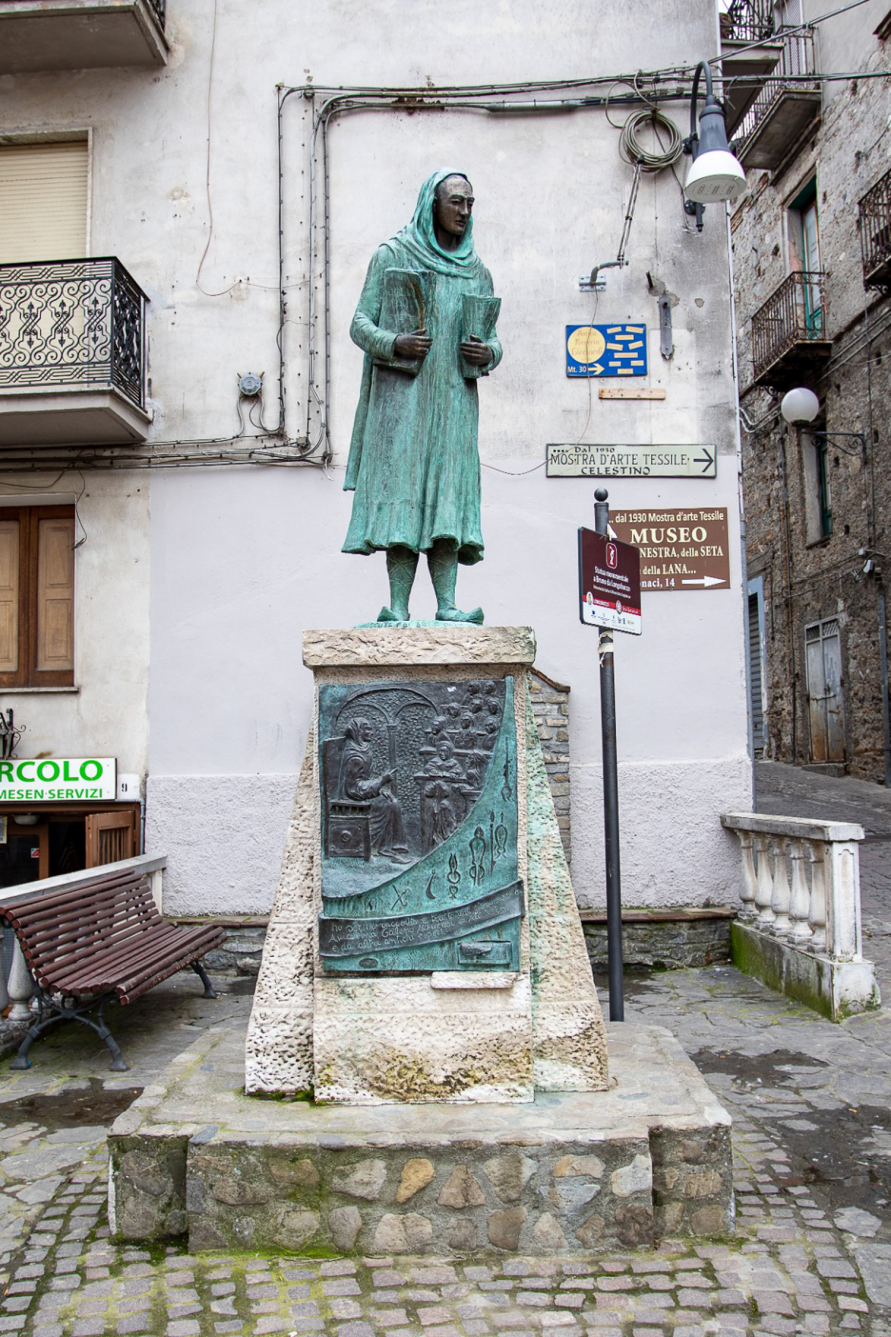
The sculpture of Bruno da Longobucco (Bruno da Longoburgo), in the free interpretation of the local artist Thomas Pirillo (1952–2021). Central Square of Longobucco town. (Courtesy of Proloco del Comune di Longobucco).

**Figure 4:**
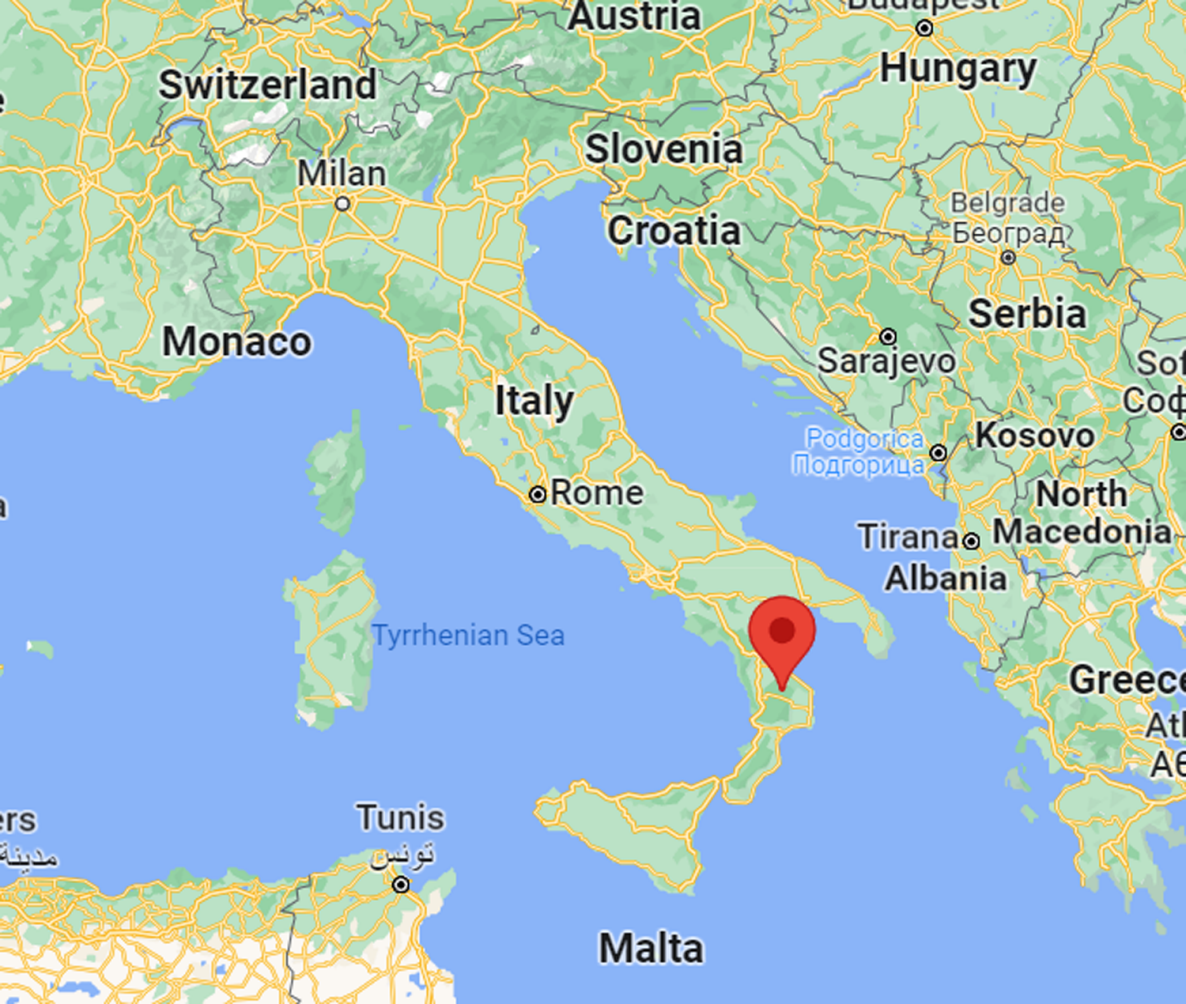
Geographic position of Longobucco, from google maps http://maps.google.com (Accessed 6 August 2022).

Indeed, as such little information exists on the life of Bruno (Figure 3), the only reliable sources are essentially his books (12). He was likely born circa 1200 in Longoburgo, today Longobucco (Figure 4), a southern Italian city in the Calabria region, whos economy flourished during the 11th–12th centuries under the Normanian-Svevian dominion (13).

Bruno probably started his education in Calabria, between Longobucco and Rossano, and then trained in medicine at the School of Salerno, at the time the most prestigious medical centre worldwide (13, 14). There are no direct records of the presence of Bruno in Salerno, but the mention of the *Articella*, a collection of Latin medical books utilized and distributed by the Salerno professors, in the introduction of his first book, “***Chirurgia Magna***” (“Greater Surgery”), together with his geographical proximity and the fact that surgery was part of medical teaching at that time in Salerno, suggests his attendance at the school of Salerno at some point of his training (15). As reported by several authors (13, 16, 17), he probably then moved to Bologna, when came in contact with Hug da Lucca and his son Theodoric, a family devoted to the practice of surgery, passed on in secret down through family generations (18).

It is in Bologna that he likely came in contact with the Arabic authors Avicenna, Albucasis, and Haly Abbas translated into Latin by Constantine the African, a Benedictine monk of the eleventh-century, and Gerard of Cremona (19). He then moved to Padua, where he was one of the founders of the University and the first lecturer of Surgery (20, 21).

**Figure 5:**
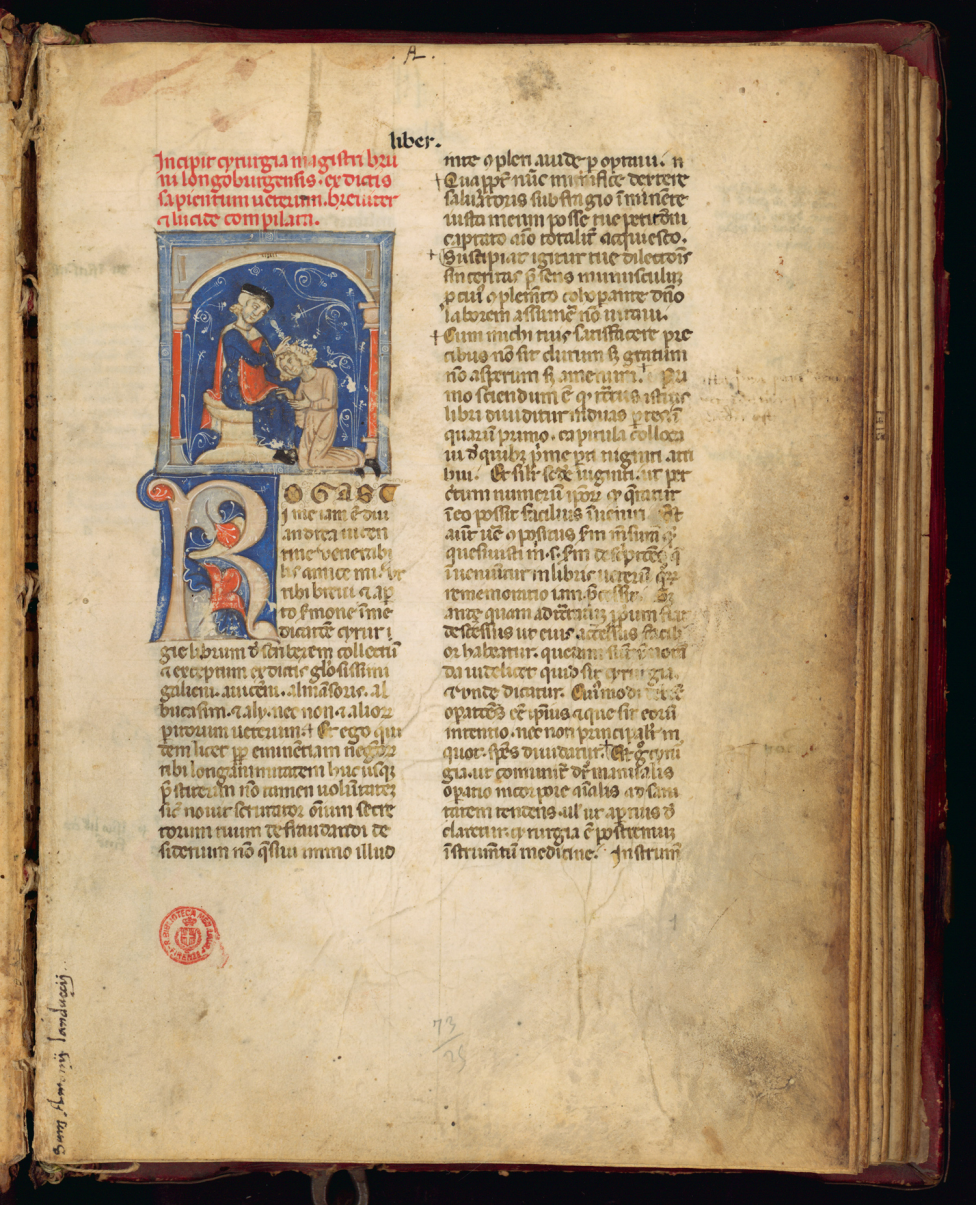
The first page of Chirurgia Magna (“Chirurgia magistri Bruni”) in a manuscript of the 14th century (from Biblioteca Medicea Laurenziana, Florence, Italy. Ms. Plut. 73.25, c. 1r, permission by Italian Ministry of Culture (Ministero della Cultura—MiC). Any reproduction without authorization with every means is prohibited.

**Figure 6:**
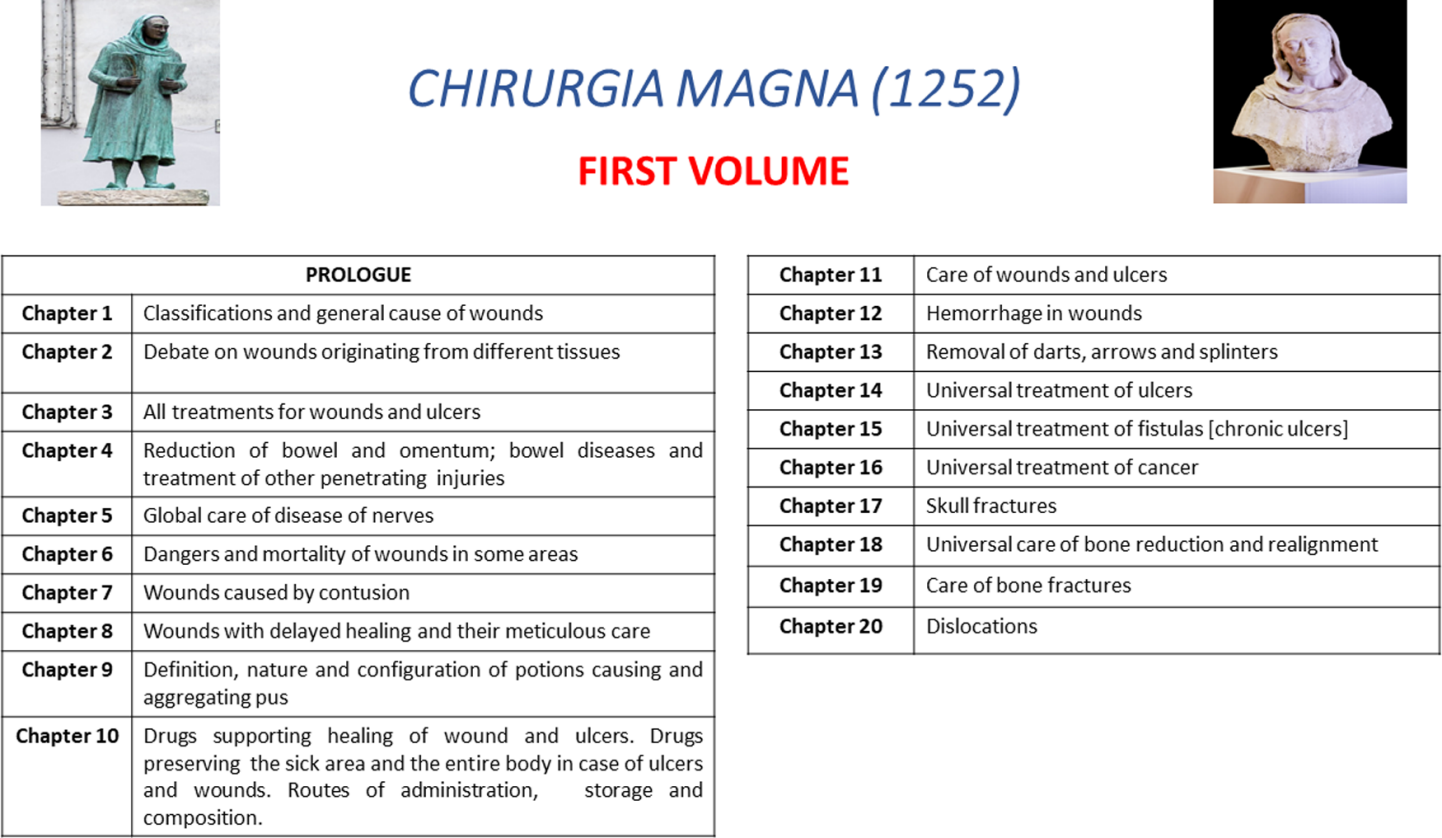
Chapters and related topics of the first volume of *Chirurgia Magna* (1252).

**Figure 7:**
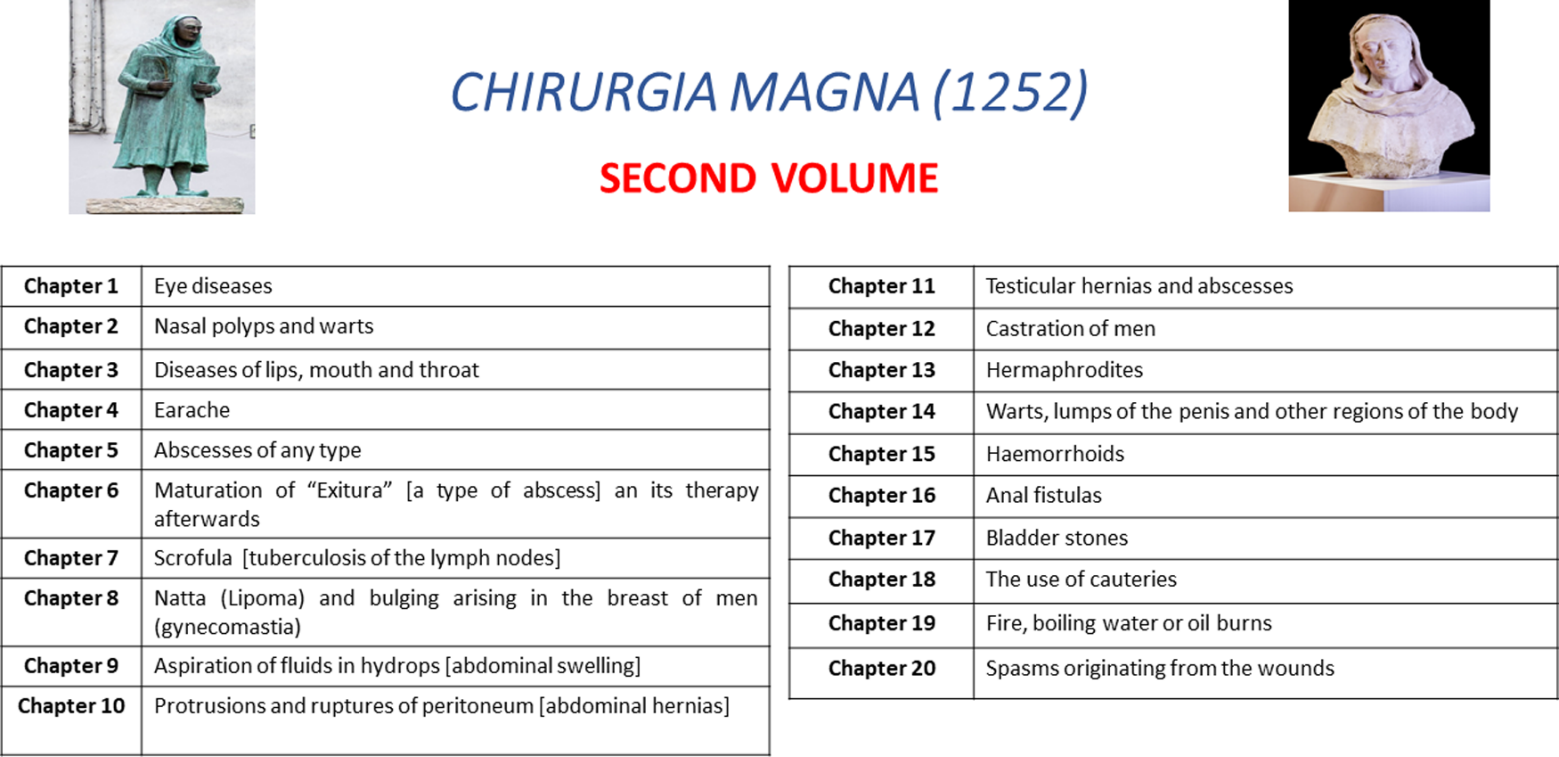
Chapters and related topics of the second volume of *Chirurgia Magna* (1252).

There, he wrote the book **Chirurgia Magna** (The Greater Surgery) in 1252 (Figure 5). The book is written in Latin, the universal scientific language of the Middle Ages, demonstrating that Bruno promoted surgery with a belief in its equity of scientific and theoretical importance with Medicine. The book is dedicated to Andrea da Vicenza, a student of Bruno in Padua. The book is divided in two volumes, each containing 10 chapters. Figures 6, 7 describe the structure of Chirurgia Magna.

The first volume is focused on wounds (chapters 1st–17th), fractures (18th–19th) and dislocations (chapter 20th). The second volume is focused on the common no-traumatic surgical topics, such as eye diseases, nasal polyps, oral diseases (chapters 1st–3rd), struma (neck lumps), scrofula (glandular swellings), lipomas, hydropsy (an ancient term to indicate any accumulation of fluid in the body) (22), hernias, castration in the men, haemorrhoids, anal fistula, the use of cautery, burns and spasm/pain originating from wounds.

In 1253, Bruno wrote ***Chirurgia Parva***, a compendium of his previous book divided into 23 chapters, where all themes are explained in a summarized version. The book is dedicated to Lazzaro of Padua (likely another student) and probably born by the need of a more efficient and cheaper reproducible text of education at a time when the printing press was yet to be invented and the cost of a manually rewritten book was highly expensive in time and resource (13).

Some authors mention other minor works by Bruno, but most of them are probably extracts of the two main works and a systematic record or study of these is missing (23). Subsequently, he probably moved to Verona and likely died there around 1286 (24).

## Original features of Chirurgia Magna

The first innovative features of Chirurgia Magna is the order of topics. The book does not follow the common order of medieval treatise “*a capite ad calcem*”, the so-called head-to-foot order. This system was commonly used to order topics easily in those times, but Bruno innovated a change to this strategy, establishing an order he considered more appropriate for the medical student/apprentice. Each theme is developed from simple lessons to more complex theories for a teaching purpose.

The introduction of the book outlines his ideas about Surgery and Surgeons. Firstly, surgery, from the Greek words, “*cheir*” (hand) and “*ergon*” (work) means “*a manual activity to heal an animal body*” and is the third arm of Medicine, to be used when the other two, diet and drugs, do not work. The aim of surgery is to “*re-join the separated things, divide those unnaturally joined and eliminate superfluous*”.

He strongly advocates acknowledging and learning the texts and teachings of classic authorities, referred as *auctoritates*, but all statements need to be accepted “*postquam certificatus sum eis, testimonio rationis et exercitio*”, only after they are validated by reason and experience. For these reasons, Bruno is claimed as a precursor of the scientific method (25).

He also advocates that experience and attendance at sites where skilled surgeons operate is necessary for training and that surgeons must be literate to distinguish themselves from “idiots and fools”. He adds the surgeon should be not alcohol addicted and possess a natural disposition toward medicine and surgery. He states surgeons should not be intrepid and temerarious, but tactful and prudent in operating, especially in delicate sites, like the brain. This is to avoid injuries produced by those operate without intellect and reason, and who ignore the cause and the name of the disease affecting the patient.

Chirurgia Magna is also the first book of surgery which cites Arabic authors (Avicenna, Albucasis, and Averroes) as authorities, making Bruno the first representative of the so-called “Arabist school” in surgery, and suggesting that he was widely read and absorbed knowledge from many sources (26).

In terms of structure, throughout his manuscript statements of classic authors are reported, but each topic is further, explored on a background of Bruno's personal experience, recounting personal episodes and comments, underpinning his views on the importance of experience and observation in the practice of surgery, a novelty in those times. As reviewed by McVaugh, “*Bruno's Chirurgia is an explicit manifesto for the kind of rational surgery—rational in its search for causes, rational in its structure, rational in its insistence that it can be read and taught from texts*” (27).

In the first volume, he focuses on meticulous description of the treatment of wounds. He distinguishes simple from complex wounds, suggesting removal of any penetrating or contaminating foreign body, arrest any bleeding in simple wounds and using potions in complex wounds to stimulate creation of *“laudable pus”* (28). He advocates that any recent wound should be sutured whenever possible, removing silk stitches after 8 days and leaving wound ends open to drain any collection. Several pages are dedicated to wound dressing and that diet that should be administered to the patient to complement wounds healing. In the chapter 6, he describes that any lesion involving bladder, brain, heart, kidney, liver, diaphragm, stomach, small bowel injuries, are usually fatal, in accordance with Hippocrates’ aphorisms (29). Finally, he demonstrates deeper knowledge and practice in the cranial trepanation, removal of arrows and splitters (reporting personal experiences) and provides a separate paragraph describing treatment for each type of dislocation.

In the second volume, he describes many other procedures (such as the drainage of abdominal cavity by an iron tube in case of ascites, “*three fingers below the umbilicus*”), already described by previous authors, but with further refinements and clarity.

Bruno da Longobucco was the first western surgeon to describe operative male castration, performed in those days often to ensure that males who were left behind to care and protect women, for example, during a war campaign, would not have an appetite to engage in sexual intercourse. He taught that the testicular excision was superior to crushing, because residual tissue remains in the latter, partially preserving their function. Here, he advocates the opinions of Paul of Aegina and Albucasis and likely strong cultural contacts between Arabic and byzantine cultures within Calabria, where there was a historical practice in using eunuchs to guard the virgins of the wealthy Arabic families (30).

He was one of the first in addressing the topic of hermaphroditism, distinguishing 4 types (3 in the men and 1 in the women) advocating surgery in three of them. He was the first Latin writer to use Arabic texts as sources on this topic, curiously not replicated by other contemporary authors (30). Regarding gynecomastia, he states the causes as excess of fat, proposing its excision by a moon-shaped incision, in a section identically reproduced later by Theodoric (31).

In relation to haemorrhoids, he distinguishes internal haemorrhoids (“*inside the anus and bleeding*”) and external haemorrhoids (“*they bleed rarely and have the same colour of the body*”). In terms of associated surgical treatment, he suggests phlebotomy as the first step and then the excision of the nodules followed by cauterization. In case of patient reluctance, the ligation by a silk thread is proposed, in accordance with Galen's previous teachings (32). When addressing anal fistula, Bruno advocates fistulotomy with a boldness of approach rare in those times (33).

Abdominal hernias are considered in his teachings as a rupture of the peritoneum (*siphac*). He states umbilical hernias should have the content ligated (not in case of involvement of bowel), then reduced and the orifice closed by stiches. In inguinal hernias, Bruno recommends reducing the hernia content and applying cautery to close the defect, also suggesting a required rest period in a supine position for 40 days and dressings to the wound area until healed.

## Influence of Bruno’s works in the Middle Ages

Chirurgia Magna was the first and leading work of medicine in the University of Padua (34) and the most consulted surgical book of the Middle Ages (13). During the Middle Ages, the study of Chirurgia Magna was mandatory in many medical schools as documented in the statutes of the Universities of Padua, Ferrara and in the Medical School of Salerno from the 13th to the 15th century (5, 8, 35). There is some indirect evidence of its use in other academic environments, such as the University of Oxford (36).

In 1270s, Jean de Saint-Amand, professor of Medicine in the University of Paris, wrote the manuscript *Revocativum Memorie*, with the intent to summarize and update all knowledge necessary for medical students. The work is divided in 4 parts, the first three wrote by Saint-Amand himself and the 4th represented by the Chirurgia Magna of Bruno, “full and unedited” as considered «*ordinata et sufficiens*» (ordered and appropriate) for students (5). Accordingly, a period of subsequent teaching of Bruno at University of Paris has been suggested (37), but never demonstrated.

Later, Bruno's books got widely disseminated in the Middle Ages: Chirurgia Magna was translated into French, Italian, German, Dutch and Hebrew (38). A fragment of a Latin edition has been also discovered in Oslo (Norway) and the existence of a translation in Catalan is documented, although lost in the 19th century (13).

The first printed edition was issued in Venice in 1498 as “La Cyrogia del Maestro Bruno” (The Surgery of Master Bruno) becoming a best-seller with five reprints by 1549 (39). Guy de Chauliac (1300–1368), one of the most important surgeons of the fourteenth century, recognized the role of Bruno as a protagonist of the renaissance of surgery in the 13th century. He cites Bruno as ***auctoritas*** (authority) 46 times in his main book “Chirurgia Magna” (1363) (2), but states that Bruno “*correctly adopted the teachings of Galen and Avicenna and the techniques of Albucasis, though he did not have a complete translation of Galen's books and left out anatomy almost entirely*”. Actually, this comment appears rather too severe, especially in view of anatomy, which became an ancillary discipline of surgery only in the 14th century. At the time of Bruno, it was mainly considered a part of natural philosophy and studied not to advance medical knowledge, but to increase the knowledge about God and creation (40).

It is notable that despite his impact, several books on the history of surgery do not mention Bruno at all (41–45), often considering the pre-eminent book to be Chirurgia (Surgery) of Theodoric, son of Hug da Lucca and bishop of Cervia, who was largely inspired by Bruno’s work, as also recorded by Guy de Chauliac.

## Conclusions

Bruno da Longobucco was the first academic surgeons of the Middle Ages. He is celebrated as one of the founders of the University of Padua and first professor of Surgery. After Roger Frugardi, he was the second author of a book of surgery in the late Middle Ages and the first in claiming the role of authorities for Arabic authors, as Avicenna, Haly Abbas and Albucasis. He enriched his books with personal episodes and observations, claiming the importance of experience and observation in the practice of surgery, a novelty in those times. He was first among western authors in describing the operation of castration for men and reported a modern approach in some fields, such in the treatment of anal fistula. Chirurgia Magna as a result was a reference text in many medical faculties for the following two centuries.

The role of Bruno has been often neglected and we hope this article may guarantee him a preserved and deserving position in respect to his instrumental role in leading an academic renaissance of surgery and delivering lasting impact in the history of surgery.
