# Corrigendum: Bruno da Longobucco (da Longoburgo): The first academic surgeon in the middle ages

**DOI:** 10.3389/fsurg.2023.1153127

**Published:** 2023-02-24

**Authors:** Francesco Pata, Cataldo Linardi, Richard R. Brady, Gianluca Pellino, Giancarlo D’Ambrosio

**Affiliations:** ^1^ Nicola Giannettasio Hospital, Corigliano-Rossano, Italy; ^2^ Sapienza University of Rome, Rome, Lazio, Italy; ^3^ Newcastle University, Newcastle Upon Tyne, North East England, United Kingdom; ^4^ University of Campania Luigi Vanvitelli, Caserta, Campania, Italy; ^5^ Vall d’Hebron University Hospital, Barcelona, Catalonia, Spain

**Keywords:** history of surgery, Bruno da Longobucco, Longoburgo, surgeon, surgery, Middle Ages, medieval, historical overview

A Corrigendum on Bruno da Longobucco (da Longoburgo): The first academic surgeon in the Middle Ages By Pata F, Linardi C, Brady RR, Pellino G and D'Ambrosio G. (2022) Front. Surg. 9:1025987. doi: 10.3389/fsurg.2022.1025987

**Text Correction**

In the published article, there was an error in the abstract, the date originally displayed for the life of Bruno da Longobucco was (1200–1286 BC) instead of the data alone without BC. A correction has been made to the Abstract. This sentence previously stated:

(1200–1286 BC)

The corrected sentence appears below:

(1200–1286)

The authors apologize for this error and state that this does not change the scientific conclusions of the article in any way. The original article has been updated.

